# Color and Spatial Cue Modulation of Emotional Word Processing: Evidence from ERPs

**DOI:** 10.3390/jintelligence14100260

**Published:** 2026-10-15

**Authors:** Xiaoyang Luo, Chao Li, Yi Sun

**Affiliations:** 1 Center for Linguistics and Applied Linguistics, Guangdong University of Foreign Studies, Guangzhou 510420, China; 2 Faculty of English Language and Culture, Guangdong University of Foreign Studies, Guangzhou 510420, China

**Keywords:** emotional metaphor, color metaphor, spatial metaphor, emotional word processing, event-related potentials, embodied cognition, Chinese

## Abstract

Emotional word processing is shaped by lexical meaning and concurrent perceptual context. This study investigated when red and green color cues and symbolic upward and downward direction cues modulate the processing of positive and negative Chinese emotional words. Thirty-four native Chinese speakers were recruited, and 27 remained in the final ERP sample after behavioral and EEG-quality exclusions. The experiment used a 2 (color: red vs. green) × 2 (direction: up vs. down) × 2 (word valence: positive vs. negative) within-subject design. Positive words elicited larger P1 amplitudes, and negative words elicited more negative-going N1 and N400 amplitudes. A significant Color × Valence interaction emerged in the late positive complex (LPC) window. The positive-minus-negative LPC difference was descriptively larger under green cues than under red cues, and neither within-color comparison reached significance after Bonferroni correction. Interactions involving spatial direction were not significant across the four components. These findings trace emotional word processing from early valence-sensitive visual attention through semantic processing to late color-dependent evaluation. The temporal pattern supports a context-sensitive account in which perceptual cues, lexical valence, and learned cultural associations contribute to emotional meaning at different processing stages.

## 1. Introduction

Emotional words convey affective states, evaluations, and socially relevant information through lexical and semantic forms. Their processing involves the recognition of written forms, access to lexical meaning, activation of affective knowledge, and evaluation of emotional significance. Positive and negative words can influence attention, memory, semantic judgments, and response selection, demonstrating that affective meaning participates in several stages of language comprehension (Kissler et al., 2006; Citron, 2012; Briesemeister et al., 2014). Emotional word processing can therefore be understood as a dynamic process in which linguistic information and affective experience jointly contribute to the construction of meaning.

This process develops within multiple forms of context. Lexical properties such as valence, arousal, concreteness, familiarity, and word frequency can influence the accessibility and evaluation of emotional meaning (Kanske & Kotz, 2007; Citron, 2012). Task requirements guide attention toward particular aspects of a word, while the surrounding semantic context shapes the information available during comprehension. Written words are also presented within a visual environment whose perceptual features can contribute to affective evaluation. Among these features, color and vertical position can carry affective meanings acquired through perceptual regularities, social conventions, and cultural experience. Colors have been systematically associated with emotional concepts and evaluations (Elliot & Maier, 2012; Jonauskaite et al., 2020), and vertical positions have been linked to positive and negative affective meanings (Meier & Robinson, 2004). Presenting emotional words together with color and spatial cues therefore provides a means of examining how lexical meaning is processed in relation to concurrent perceptual context.

Temporal evidence from event-related potential research has further clarified how emotional information becomes available during word processing. Emotional word effects have been observed during early visual and attentional processing, semantic access, and later evaluative processing (Herbert et al., 2008; Scott et al., 2009; Citron, 2012; D. Zhang et al., 2014). Early components such as P1 and N1 can reflect sensitivity to visual and affective information, and later components such as N400 and LPC provide measures of semantic and sustained evaluative processing (Bayer et al., 2012; Kutas & Federmeier, 2011; Kuchinke et al., 2015). Recent studies continue to show temporally distributed neural sensitivity to emotional information conveyed by written words, including evidence from Chinese word processing (Juuse et al., 2023; K. Zhang et al., 2024). ERP measures therefore provide a suitable method for tracing when perceptual context participates in emotional word processing.

Language and cultural experience provide a further context for the organization of emotional meaning. Cross-linguistic research shows that emotion semantics contain broadly shared patterns together with systematic variation associated with linguistic and cultural experience (Jackson et al., 2019). Spatial metaphors of affect also vary across cultural communities, reflecting locally established ways of relating emotional experience to the body and surrounding space (Wnuk & Ito, 2021). Color-emotion associations show comparable cultural patterning, with linguistic and geographic proximity contributing to the meanings associated with particular colors (Jonauskaite et al., 2020). Studies conducted within specific languages have consequently provided important evidence about the relationship between shared perceptual experience and culturally established meaning. Research on Chinese has documented embodied patterns in conceptual organization (Yu, 1998), multiple cultural meanings associated with red in the Sinophone world (Bogushevskaya, 2024), language-specific semantic extensions of Chinese HONG (Song & Wang, 2026), and electrophysiological sensitivity to the emotional connotations of Chinese words (D. Zhang et al., 2014; K. Zhang et al., 2024). These findings establish the value of investigating emotional word processing within a Chinese linguistic and cultural setting. The present study uses ERPs to examine how red and green color cues and upward and downward spatial cues modulate the temporal processing of positive and negative Chinese nouns.

## 2. Literature Review

Research on emotional word processing has identified spatial orientation and color as perceptual dimensions associated with affective meaning. Behavioral studies have illustrated how these dimensions influence judgments and response times, and ERP studies have traced their influence across early perceptual, semantic, and later evaluative stages.

Behavioral evidence has consistently documented associations between emotional valence and vertical space. Meier and Robinson (2004) found that positive words were evaluated more efficiently in upper spatial locations and negative words in lower locations. Subsequent studies extended this association across participant groups and three-dimensional spatial arrangements (Marmolejo-Ramos et al., 2017, 2018). Cross-cultural research has also shown that spatial metaphors of affect draw on shared bodily experience and culturally conventionalized patterns (Wnuk & Ito, 2021). A recent meta-analysis reported a reliable overall vertical space-valence association, with positive concepts linked to upper space and negative concepts to lower space, while also identifying substantial between-study heterogeneity (Pohl & Miklashevsky, 2025). These findings show that vertical position can participate in the organization and evaluation of affective concepts.

Electrophysiological studies provide additional evidence concerning the time course of spatial-conceptual associations. Zanolie et al. (2012) reported behavioral and ERP evidence that abstract social concepts can shift attention along a vertical dimension. Xie et al. (2014) observed ERP differences associated with valence-space pairings during affective word processing, and Y. Zhang et al. (2015) found that arrow direction and emotional valence influenced P2, N400, and LPC responses. Arrow direction functions as a symbolic representation of vertical space and can also guide visual attention. Symbolic arrow cues can produce attentional orienting even when they provide no predictive information about a subsequent target location (Hommel et al., 2001; Tipples, 2002). Arrow direction therefore provides a spatial cue that can engage learned valence-space associations and visual orienting processes during word processing.

Color also carries evaluative and emotional meaning. Color-in-context theory proposes that color meanings develop through repeated associations with particular objects, situations, and actions, with the current context guiding their psychological influence (Elliot & Maier, 2012; Elliot, 2015). Red has been associated with warning, error, danger, heightened arousal, and attentional capture in several task settings (Kuniecki et al., 2015; Fetterman et al., 2019; Xia et al., 2018). Green has frequently been associated with nature, safety, growth, and vitality (Clarke & Costall, 2008; Elliot & Maier, 2012). Experimental studies further show that color can influence emotional evaluation, memory, and attention (Kuhbandner & Pekrun, 2013; Gan et al., 2016). Color can participate in emotional processing through associations established within specific objects, tasks, and situations.

Cultural experience further shapes color-emotion associations. Large-scale cross-cultural evidence shows widespread regularities in color-emotion mappings together with systematic variation related to language and geographic proximity (Jonauskaite et al., 2020). In Chinese cultural contexts, red is associated with celebration, good fortune, success, prosperity, warning, and danger. Research on visual and linguistic representations of red in the Sinophone world documents the historical and cultural development of these meanings (Bogushevskaya, 2024). Song and Wang (2026) compared the semantic profiles of Chinese HONG and English RED and identified shared experiential foundations and language-specific semantic extensions. Green is commonly connected with nature, safety, and vitality, and its evaluative meaning also develops within particular cultural and situational settings. These findings identify color as a context-sensitive cue whose relationship with emotional valence can be discovered directly in Chinese emotional word processing.

ERP research has identified early sensitivity to emotional information during visual word processing. The P1 is associated with early visual encoding, stimulus salience, and initial attentional allocation, and the N1 is associated with perceptual discrimination and selective attention (Luck et al., 1990; Bayer et al., 2012). Emotional valence can modulate early responses to written words, indicating that affective information becomes available during early stages of visual processing (Scott et al., 2009; D. Zhang et al., 2014). Evaluative conditioning of positive and negative word valence has also been found to affect P1 and N1 amplitudes (Kuchinke et al., 2015). Recent evidence concerning Chinese monomorphic and compound words further demonstrates early electrophysiological sensitivity to emotional connotations (K. Zhang et al., 2024). P1 and N1 therefore provide temporal measures of early perceptual and attentional sensitivity during emotional word processing.

Later components provide information about semantic and evaluative processing. The N400 is sensitive to semantic access, expectancy, and the relationship between a word and its processing context (Kutas & Federmeier, 2011; Van Petten & Luka, 2012). Emotional valence, semantic organization, task demands, and contextual information can contribute to N400 variation during word processing (Kanske & Kotz, 2007; Citron, 2012; Gan et al., 2016). The late positive complex (LPC) is associated with sustained attention, affective evaluation, elaborative processing, and the continued use of contextual information (Herbert et al., 2008; Hajcak et al., 2010; Citron, 2012; Li et al., 2013). Recent ERP evidence also confirms temporally distributed sensitivity to emotional information conveyed by words (Juuse et al., 2023). Together, P1, N1, N400, and LPC provide a temporal sequence for examining perceptual, semantic, and evaluative processes during emotional word processing.

## 3. Theoretical Framework

Embodied cognition provides the general cognitive basis for relating perceptual experience to emotional meaning. Embodied accounts propose that conceptual knowledge is grounded in interactions among perception, action, bodily states, and the environment (Varela et al., 1991; Barsalou, 1999; Wilson, 2002). Perceptual simulation allows language comprehension to recruit experience-based representations associated with conceptual content (Gallese & Lakoff, 2005; Bergen, 2012). Emotional concepts draw on internal affective states and recurrent patterns in the external environment. Vertical direction and color can therefore contribute experiential information through which emotional meaning is organized and processed.

Conceptual metaphor theory (CMT) specifies how abstract concepts are structured through systematic mappings from concrete domains of experience (Lakoff & Johnson, 1980, 1999). Within the emotional domain, perceptual dimensions such as verticality, color, temperature, force, and bodily movement can provide experiential structures for organizing affective meaning (Kövecses, 1991, 2000; Yu, 1998). Associations between positive affect and upward space, negative affect and downward space, and culturally acquired color meanings illustrate how recurring experience contributes to the conceptual organization of emotion. Building on CMT, the Extended Conceptual Metaphor Theory (ECMT) broadens the account of meaning construction across image schemas, domains, frames, and mental spaces (Kövecses, 2015, 2020). Current discourse, bodily experience, situational information, and cultural knowledge guide the activation of relevant conceptual structures. This development from CMT to ECMT provides a framework for examining how spatial direction, color, cultural knowledge, and immediate task context jointly contribute to the temporal processing of emotional words.

These theoretical perspectives contribute to the present study at complementary explanatory levels. Embodied cognition provides the general proposition that perceptual and bodily experience can participate in emotional meaning processing. CMT specifies systematic conceptual associations through which vertical direction and color can become linked with emotional valence. ECMT further predicts that the online contribution of these associations is shaped by cultural knowledge, the immediate task context, and the configuration of concurrently available cues. In the present ERP design, valence effects provide evidence about the temporal processing of emotional word meaning. Interactions between word valence and color or direction indicate context-dependent modulation associated with the perceptual cues. The timing of these interactions provides evidence about when perceptual context enters visual, semantic, and evaluative processing.

Within this framework, emotional metaphor refers to systematic conceptual associations linking emotional valence with perceptual dimensions such as vertical direction and color. The present experiment operationalized these associations through factorial combinations of positive and negative Chinese nouns with red or green arrows pointing upward or downward. Color cue refers to the chromatic property of the arrow, and spatial cue refers to its vertical direction. Emotional word processing was assessed through behavioral task performance and mean amplitudes in the P1, N1, N400, and LPC time windows. Effects involving color or arrow direction provide evidence concerning perceptual-cue modulation during emotional word processing and can be evaluated in relation to embodied and context-sensitive theoretical accounts.

Existing research has established associations between emotional valence and vertical space, context-sensitive associations between emotional evaluation and color, and successive ERP stages in emotional word processing. Embodied cognition, CMT, and ECMT connect these findings through perceptual experience, conceptual association, cultural knowledge, and current context. Previous experiments have primarily revealed spatial and color cues in separate paradigms. Their simultaneous contribution to Chinese emotional word processing remains. Evidence concerning the temporal organization of these influences can clarify how concurrent perceptual cues participate in early visual processing, semantic processing, and later evaluation. The multiple cultural meanings of red also create a specific need to examine color effects within the present Chinese language and task context.

Building on abovementioned evidence and the theoretical development from CMT to ECMT, the present study investigated how color and spatial cues modulate the temporal processing of positive and negative Chinese words. A 2 × 2 × 2 within-subject design crossed word valence, arrow color, and arrow direction. The study addressed three research questions: (1) How do color cues modulate the temporal processing of positive and negative Chinese words? (2) How do vertical spatial cues modulate the temporal processing of positive and negative Chinese words? (3) How are the influences of color and spatial cues organized when both cues are presented simultaneously? Addressing these questions can clarify the temporal organization of perceptual-cue modulation during emotional word processing and provide evidence for evaluating context-sensitive embodied accounts.

## 4. Materials and Methods

### 4.1. Participants

Thirty-four healthy adult participants were recruited from a university in South China (18 females, 16 males; mean age = 19.77 years, SD = 1.62). The recruitment target was informed by sample sizes used in previous within-subject ERP studies of emotional valence and spatial cue processing (Xie et al., 2014; Y. Zhang et al., 2015). All participants were native Chinese speakers and right-handed, reported normal or corrected-to-normal vision, and had no color blindness, color vision deficiency, or reading impairment. None had previously participated in a similar spatial-color cue or emotional word judgment experiment. One participant was excluded because overall task accuracy was below 90%, and six additional participants were excluded because their recordings did not provide sufficient artifact-free EEG data for reliable ERP averaging. The final ERP sample comprised 27 participants. The experimental procedures were approved by the Bilingual Cognition and Development Lab Ethics Committee, Guangdong University of Foreign Studies (approval date: 22 June 2025; approval No. BCD-20250603). Written informed consent was obtained before the experiment. Participants were informed of their right to withdraw at any time and received compensation after completing the task.

### 4.2. Materials

#### 4.2.1. Emotional Word Stimuli

Emotional word stimuli were selected from the Chinese Affective Words System (CAWS; Y. Wang et al., 2008). The final set comprised 80 positive and 80 negative two-character Chinese nouns. Selection was based on valence and arousal ratings provided by CAWS on a 9-point scale. Positive words had a mean valence rating of 6 or higher, and negative words had a mean valence rating of 4 or lower. Mean arousal ratings for both categories were 5.4 or higher. Positive words had higher valence ratings than negative words. Familiarity and concreteness were assessed in a separate pretest involving 20 raters who did not participate in the ERP experiment. Each rater evaluated all selected words using 9-point scales consistent with the CAWS rating format. Familiarity ratings ranged from 1 (very unfamiliar) to 9 (very familiar), and concreteness ratings ranged from 1 (highly abstract) to 9 (highly concrete). The positive and negative word sets were matched on arousal, stroke count, log-transformed word frequency, familiarity, and concreteness. Word frequency values were obtained from the Beijing Language and Culture University Corpus (Zhan et al., 2019), and stroke counts were retrieved from the Online Xinhua Dictionary. Descriptive statistics and matching results are shown in Table 1.

#### 4.2.2. Color and Spatial Cues

Color and spatial information were presented through compound arrow cues. Arrow hue operationalized the color cue (red or green), and arrow orientation operationalized the symbolic vertical-direction cue (upward or downward). Each display contained two arrows with the same hue and orientation and an emotional word at the center of the screen. Stimuli were presented on a gray background. The programmed RGB values were red = (207, 20, 14), green = (61, 195, 114), and gray = (134, 134, 134). Approximate relative luminance values calculated from these sRGB values were 0.14 for red, 0.41 for green, and 0.24 for gray. The absolute Michelson contrast against the gray background was approximately 0.27 for both cue colors. Red and green were selected as visually discriminable colors with established context-dependent affective associations (Clarke & Costall, 2008; Elliot & Maier, 2012; Fetterman et al., 2019; Jonauskaite et al., 2020). Upward and downward arrows represented the vertical dimension examined in previous valence-space research (Meier & Robinson, 2004; Zanolie et al., 2012; Y. Zhang et al., 2015).

### 4.3. Experimental Design

The experiment used a 2 (color cue: red vs. green) × 2 (spatial cue: up vs. down) × 2 (word valence: positive vs. negative) within-subject design, yielding eight experimental conditions. Color and direction were cue-level factors, and valence was a lexical target factor. Each participant completed 40 experimental trials in each condition before behavioral and EEG trial rejection.

The Color × Valence interaction assessed whether emotional word processing varied across red and green cues. The Direction × Valence interaction assessed whether emotional word processing varied across upward and downward cues. The Color × Direction × Valence interaction assessed whether the relationship between either cue dimension and word valence depended on the concurrent cue. The simultaneous compound-cue arrangement followed the general logic of affective interference paradigms in which lexical and visual information are presented together (Stenberg et al., 1998; Zhu et al., 2010).

### 4.4. Procedure

The experiment was programmed in E-Prime 3.0 (Psychology Software Tools, Inc., Sharpsburg, PA, USA) and conducted in the Bilingual Cognition and Development Laboratory at Guangdong University of Foreign Studies. Stimuli were presented on a 24-inch monitor with a 60 Hz refresh rate, and participants were seated approximately 80 cm from the screen in a sound-attenuated room. EEG signals and E-Prime event markers were synchronized throughout the experiment. Participants were instructed to minimize eye movements, blinks, and body movements during recording.

**Figure 1:**
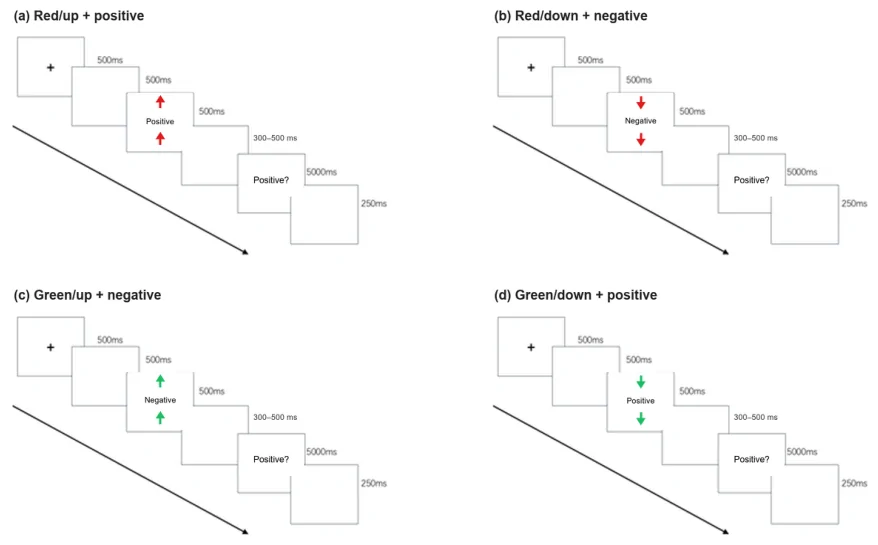
Experimental trial presentation. English labels are used in the schematic for readability; participants viewed Chinese words and Chinese prompts. Red and green indicate the color cue, and upward and downward arrows indicate the spatial-direction cue.

**Figure 2:**
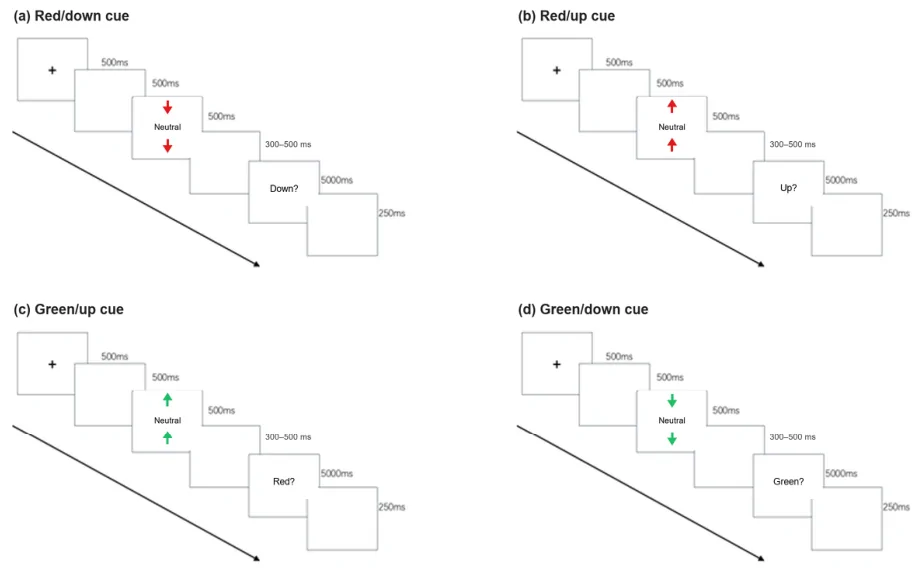
Filler trial presentation. English labels are used in the schematic for readability; participants viewed Chinese words and Chinese prompts. The response prompt queried either arrow color or arrow direction.

Before the formal experiment, participants completed a 10-trial practice block containing four positive words, four negative words, and two filler words. Practice materials did not appear in the formal task, and the block was repeated when additional practice was needed. Each formal trial began with a fixation cross for 500 ms, followed by a blank screen for 500 ms. A compound display containing two arrows and a central word then appeared for 500 ms. After a jittered blank interval of 300–500 ms, a question screen remained visible until a response was made or the 5000 ms response window elapsed. Each trial ended with a 250 ms blank screen. The experimental and filler trial sequences are shown in Figure 1 and Figure 2, respectively.

In experimental trials, participants judged whether the word was positive or negative. In filler trials, they judged either the color or the direction of the arrows. Responses were made with the F and J keys, and response mapping was counterbalanced across participants. The 160 emotional words (80 positive and 80 negative) were each presented twice, producing 320 experimental trials and 40 trials in each of the eight conditions. Eighty neutral filler words with arousal ratings below 5 and valence ratings between 4 and 6 were each presented once. Each participant therefore completed 400 trials. A Latin square procedure counterbalanced the assignment of emotional words to cue combinations across participants. The task was divided into blocks, with a short break after every 80 trials, and the complete session lasted approximately 1.5 h.

Experimental and filler trials were randomly intermixed, and the relevant response dimension was revealed after the compound stimulus had disappeared. At stimulus onset, participants could not predict whether the subsequent judgment would concern word valence, cue color, or cue direction. This procedure reduced potential anticipation effects. Color and direction remained task-relevant across the experiment, and neither dimension was directly judged on the experimental trials included in the ERP analysis. Filler trials were excluded from the primary ERP averages.

### 4.5. EEG Recording

EEG data were recorded continuously using a 64-channel Neuroscan system with a SynAmps2 amplifier (Compumedics Neuroscan, Charlotte, NC, USA). Electrode placement followed the international 10–20 system, and bilateral mastoids served as reference electrodes. The ground electrode was positioned at the midline of the forehead. Vertical electrooculogram (VEOG) activity was recorded from electrodes above and below the left eye, and horizontal electrooculogram (HEOG) activity was recorded from electrodes at the outer canthi of both eyes. Electrode impedances were maintained below 5 kΩ. EEG and EOG signals were sampled at 1000 Hz, and a 0.05–100 Hz band-pass filter was applied during acquisition.

### 4.6. EEG Preprocessing and ERP Quantification

Offline preprocessing was performed in MATLAB R2020b (The MathWorks, Inc., Natick, MA, USA) using EEGLAB v2021.0 (Swartz Center for Computational Neuroscience, University of California San Diego, La Jolla, CA, USA; Delorme & Makeig, 2004). Non-EEG channels (CB1, CB2, EKG, EMG, VEO, and HEO) were removed. A 0.1–40 Hz band-pass filter and a 48–52 Hz notch filter were applied, followed by downsampling to 500 Hz (Widmann et al., 2015). Only experimental trials with correct behavioral responses were epoched from 200 ms before to 1000 ms after compound-stimulus onset, and the 200 ms pre-stimulus interval was used for baseline correction. Epochs containing visible movement, muscle, or high-amplitude noise were removed before independent component analysis. Extended Infomax ICA was conducted after principal component analysis reduced the data to 50 components (Makeig et al., 1996). Components associated with eye movements, cardiac activity, and muscle activity were identified by visual inspection and removed (Jung et al., 2000). A final ±100 μV threshold was applied across channels. Across participants, the mean retention rate for experimental trials was 74.17% (SD = 13.47%), with individual retention rates ranging from 48.12% to 97.50%. When retained experimental trials were averaged across the eight conditions, participants contributed an average of 29.67 trials per condition (SD = 5.39; participant-level range = 19.25–39.00), from an initial 40 trials per condition.

ERP waveforms were averaged separately for each participant in the eight conditions. Mean amplitude within each component-specific time window and electrode region served as the primary ERP measure. P1 was measured from 130 to 170 ms and N1 from 180 to 220 ms over P3, P4, Pz, O1, O2, and Oz, following research on early visual and affective word processing (Luck et al., 1990; Bayer et al., 2012; Kuchinke et al., 2015). N400 was measured from 300 to 500 ms over F1, Fz, F2, FC1, FCz, FC2, C1, Cz, and C2 (Kutas & Federmeier, 2011; Van Petten & Luka, 2012). LPC was measured from 500 to 800 ms over Pz, CP1, CPz, CP2, P1, P2, and Cz (Hajcak et al., 2010; Citron, 2012). The component windows and electrode regions were selected from the relevant ERP literature together with the morphology and scalp distribution of the grand-average waveforms.

### 4.7. Statistical Analysis

Behavioral data were used to assess task performance and to screen trials and participants. Responses shorter than 200 ms or longer than 4999 ms were excluded, and reaction times were summarized for correct responses.

ERP analyses were conducted in R 4.5.2 (R Foundation for Statistical Computing, Vienna, Austria). Repeated-measures ANOVAs were conducted respectively for P1, N1, N400, and LPC. Each model included Color (red vs. green), Direction (up vs. down), Valence (positive vs. negative), and Electrode as within-subject factors. The Color × Valence, Direction × Valence, and Color × Direction × Valence effects addressed cue-dependent modulation of emotional word processing. Effects involving Electrode described variation within each component-specific region. Greenhouse-Geisser corrections were applied to effects with more than one numerator degree of freedom when sphericity was violated. The alpha level for each component-level omnibus model was .05. Component-wise ANOVAs were planned for four theoretically motivated ERP windows because P1, N1, N400, and LPC represent temporally and functionally distinct measures. Each component-level omnibus model was evaluated at an alpha level of .05, and effects were interpreted within the time window and electrode region defined for that component. The component-level omnibus models retained this alpha level without an additional across-component adjustment.

Significant interactions were followed by simple-effects comparisons with Bonferroni correction. ANOVA effects were quantified with partial eta squared (η^2^p), and paired comparisons were quantified with Cohen’s dz. Exact *p* values and 95% confidence intervals were reported where applicable.

## 5. Results

### 5.1. Behavioral Performance

Behavioral performance was summarized for the final 27-participant ERP sample after the participant- and trial-level screening criteria were applied. Overall accuracy was 96.16% (SD = 2.65%), and the mean reaction time for correct responses was 1133.36 ms (SD = 251.35). For positive words, mean accuracy was 97.36% (SD = 2.08%) and mean reaction time was 1081.92 ms (SD = 246.36). For negative words, mean accuracy was 94.95% (SD = 3.73%) and mean reaction time was 1186.61 ms (SD = 263.61).

### 5.2. ERP Results

**Figure 3:**
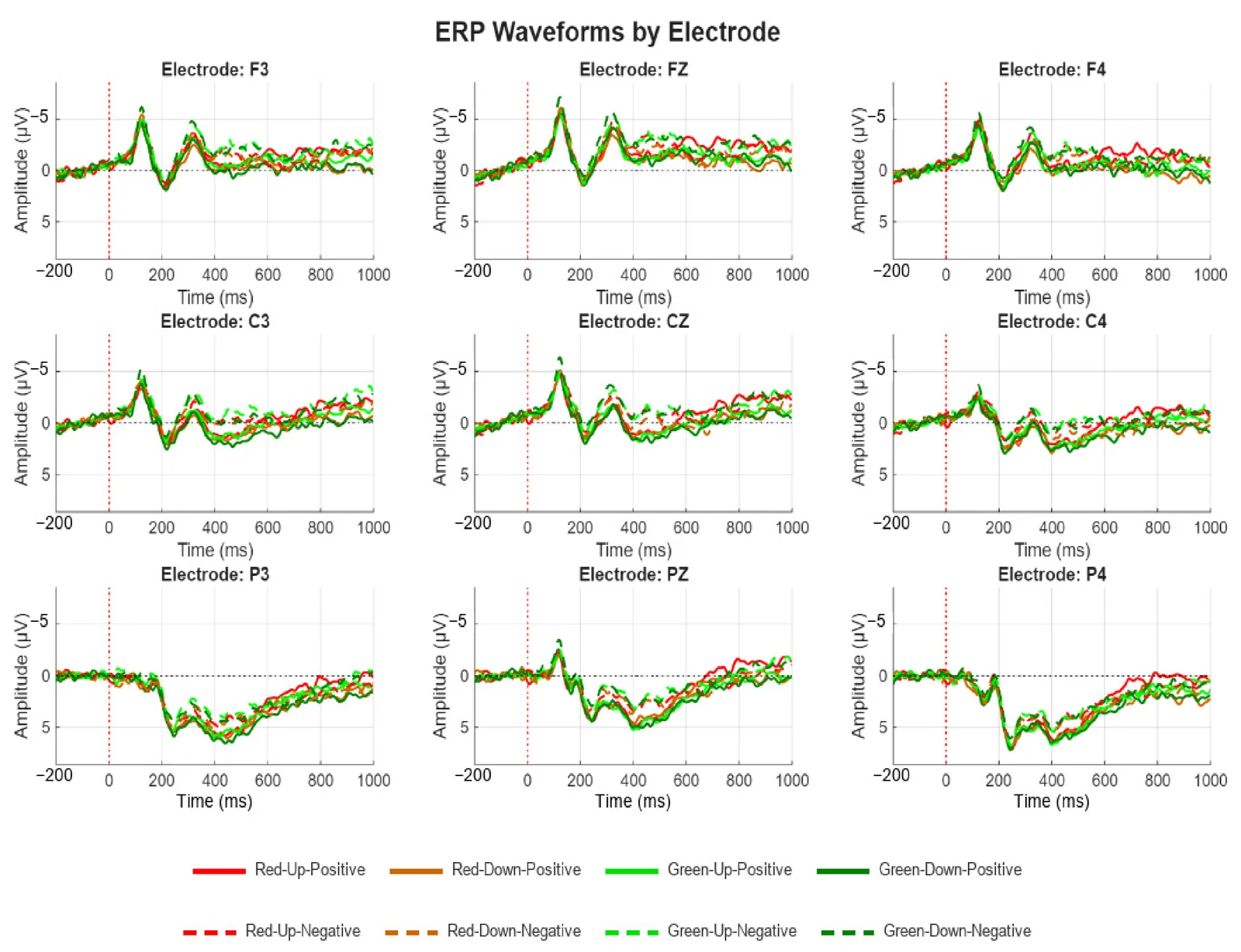
Grand-average ERP waveforms for the eight experimental conditions.

**Figure 4:**
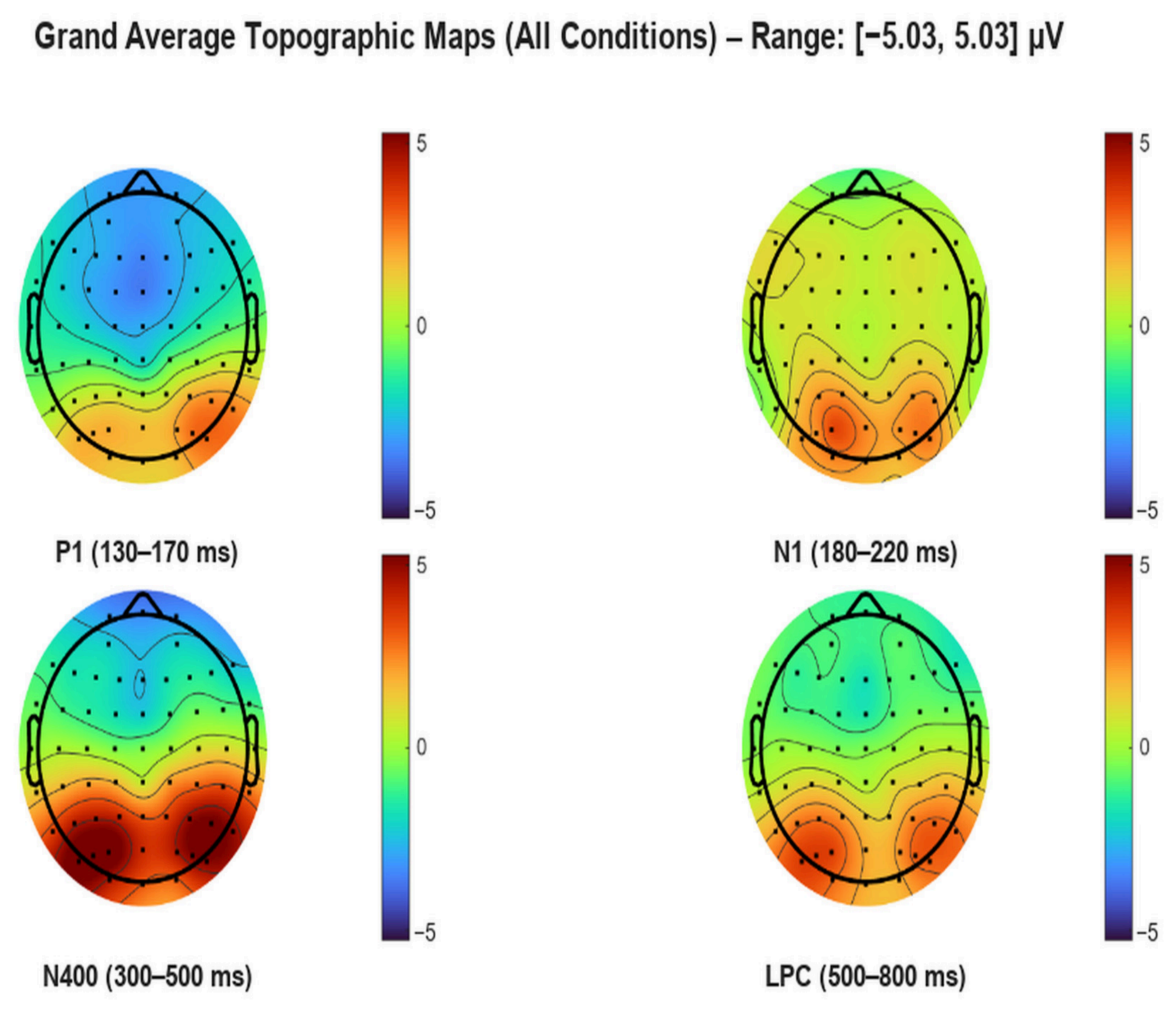
Topographic maps for the P1, N1, N400, and LPC components.

Mean amplitudes were analyzed separately for P1, N1, N400, and LPC. Word valence produced main effects in the P1, N1, and N400 windows. The LPC window showed a Color × Valence interaction. Neither the Direction × Valence interaction nor the Color × Direction × Valence interaction reached significance in any component. Grand-average waveforms and component topographies are shown in Figure 3 and Figure 4.

#### 5.2.1. P1

Positive words elicited larger mean P1 amplitudes (M = 1.43 μV, SE = 0.35) than negative words (M = 0.99 μV, SE = 0.31), yielding a main effect of valence (F(1, 26) = 4.48, *p* = .044, η^2^p = .147). The Color × Valence interaction (F(1, 26) = 1.14, *p* = .296, η^2^p = .042), the Direction × Valence interaction (F(1, 26) = 0.16, *p* = .690, η^2^p = .006), and the Color × Direction × Valence interaction (F(1, 26) = 1.61, *p* = .216, η^2^p = .058), were not significant.

#### 5.2.2. N1

Negative words elicited more negative-going mean N1 amplitudes (M = 1.80 μV, SE = 0.38) than positive words (M = 2.13 μV, SE = 0.41), yielding a main effect of valence (F(1, 26) = 6.86, *p* = .015, η^2^p = .209). The Color × Valence interaction (F(1, 26) = 0.13, *p* = .719, η^2^p = .005), the Direction × Valence interaction (F(1, 26) = 0.04, *p* = .837, η^2^p = .002), and the Color × Direction × Valence interaction (F(1, 26) = 0.32, *p* = .576, η^2^p = .012), were not significant.

#### 5.2.3. N400

Negative words elicited larger negative-going mean N400 amplitudes from 300 to 500 ms (M = −2.32 μV, SE = 0.28) than positive words (M = −1.32 μV, SE = 0.24), yielding a main effect of valence (F(1, 26) = 35.45, *p* < .001, η^2^p = .577). The Color × Valence interaction (F(1, 26) = 0.80, *p* = .379, η^2^p = .030), the Direction × Valence interaction (F(1, 26) = 0.22, *p* = .641, η^2^p = .008), and the Color × Direction × Valence interaction (F(1, 26) = 0.33, *p* = .570, η^2^p = .013), were not significant.

#### 5.2.4. LPC

**Figure 5:**
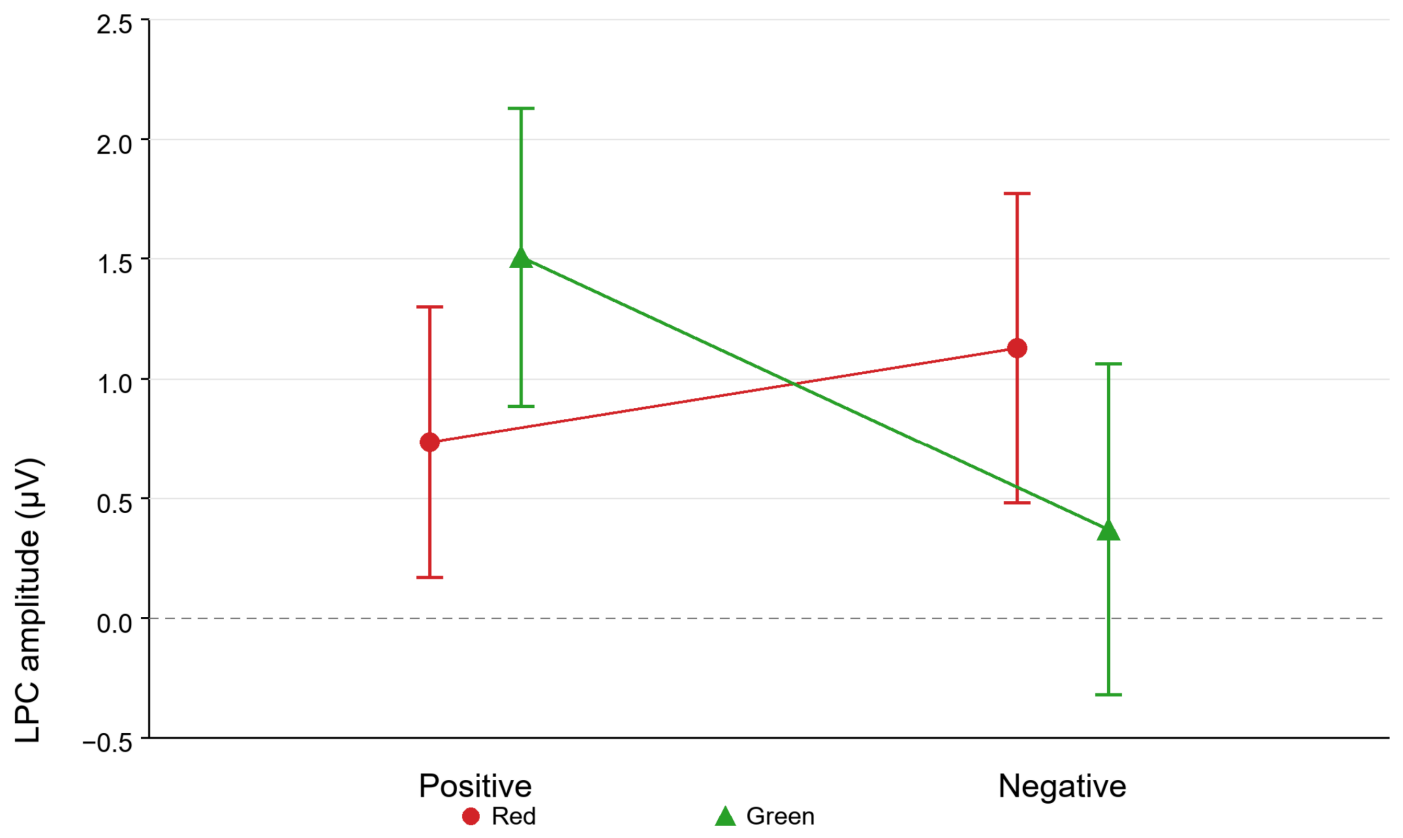
Color × Valence interaction in mean LPC amplitude during the 500–800 ms window. Error bars represent standard errors of the mean across participants (n = 27).

The Color × Valence interaction was significant in the 500–800 ms LPC window (F(1, 26) = 6.18, *p* = .020, η^2^p = .192) (Figure 5). The main effects of color, direction, and valence were not significant. The Direction × Valence interaction (F(1, 26) = 0.13, *p* = .718, η^2^p = .005), and the Color × Direction × Valence interaction (F(1, 26) = 0.02, *p* = .895, η^2^p = .001), were also not significant.

Bonferroni-adjusted valence comparisons within each color condition described the interaction pattern. Under green cues, positive words (M = 1.51 μV, SD = 3.24) showed larger mean LPC amplitudes than negative words (M = 0.37 μV, SD = 3.59), t(26) = 2.20, adjusted *p* = .074, dz = 0.42. Under red cues, positive words (M = 0.73 μV, SD = 2.93) and negative words (M = 1.13 μV, SD = 3.36) showed a smaller difference, t(26) = −1.09, adjusted *p* = .573, dz = −0.21. Neither comparison met the Bonferroni-adjusted significance criterion. Expressed as a within-subject difference-of-differences contrast, the Color × Valence interaction corresponded to a comparison of the mean of the green-positive and red-negative conditions with the mean of the green-negative and red-positive conditions. This contrast yielded a mean difference of 0.76 μV, 95% CI [0.13, 1.40], t(26) = 2.49, *p* = .020, dz = 0.48.

## 6. Discussion

The present study investigated how color and symbolic vertical-direction cues modulated the processing of positive and negative Chinese emotional words. Word valence influenced the P1, N1, and N400 components, showing a temporal progression from early visual-attentional processing to lexical-semantic processing. A significant Color × Valence interaction emerged in the LPC window, indicating that the relation between color cues and word valence became evident during later evaluative processing. Interactions involving spatial direction were not significant across the four components. These findings reveal an early-to-late sequence in which lexical valence was registered rapidly and color-dependent contextual modulation emerged at a later processing stage.

The P1 and N1 effects show that emotional valence influenced early stages of visual word processing. Positive words elicited larger P1 amplitudes, a pattern consistent with rapid allocation of perceptual attention to affectively salient lexical information (Bayer et al., 2012; Scott et al., 2009). Negative words elicited more negative-going N1 amplitudes, indicating valence-sensitive differentiation during early visual discrimination and selective attention (Luck et al., 1990; Carretié, 2014). Related studies have also located emotional-word effects within early ERP intervals and linked their magnitude to lexical and affective properties (Briesemeister et al., 2014; Kuchinke et al., 2015). The absence of cue-related interactions in these windows places the reliable color-dependent modulation later in the processing sequence. Early sensitivity to lexical valence therefore provided the perceptual and attentional conditions from which subsequent contextual evaluation developed.

In the N400 window, negative words elicited larger negative amplitudes than positive words. The N400 is sensitive to the activation and integration of meaning in relation to lexical knowledge, semantic expectancy, and the current processing context (Kutas & Federmeier, 2011). Emotional word studies have similarly shown that valence can modulate N400 activity during lexical-semantic processing (Kanske & Kotz, 2007; Herbert et al., 2008; Citron, 2012). The larger response to negative words in the present study may therefore reflect increased demands on semantic activation and contextual updating during the evaluation of negative lexical meaning. Several properties of the stimuli and task may have contributed to this valence effect. Emotionally salient words can attract processing resources and activate extensive semantic and memory associations, both of which can influence activity in the N400 interval (Kissler et al., 2006; Citron, 2012). Emotional words can also differ in the directness with which they refer to an emotional state. X. Wang et al. (2019) found that emotion-label and emotion-laden words showed different effects during the earlier P2 interval, and that both word types elicited smaller N400 amplitudes than neutral words. Their findings indicate that the organization of emotional meaning can influence the time course of word processing and that emotional semantic information from both word types contributes to N400 activity. The present positive–negative comparison further shows that N400 differentiation can occur within emotional vocabulary. The matching procedure equated the two valence categories on arousal, word frequency, stroke count, familiarity, and concreteness. Associative structure, motivational salience, and the directness of emotional reference provide additional lexical-semantic dimensions that may contribute to the observed amplitude pattern. The randomized sequence of positive, negative, and filler trials created variable trial-to-trial semantic contexts and required continual updating of the currently relevant representation. The valence-judgment task also directed attention toward emotional meaning, consistent with evidence that task requirements influence the timing and magnitude of emotion-related ERP activity (Kanske & Kotz, 2007; Bayer et al., 2012). These lexical, emotional, and task-related influences provide a plausible account of the N400 valence effect.

The central cue-related finding was the significant Color × Valence interaction in the LPC window. The LPC and related late positive activity have been associated with sustained attention, affective evaluation, and elaborative processing of motivationally relevant information (Hajcak et al., 2010; Citron, 2012). Emotional language studies further show that late positive activity is sensitive to the relevance of emotional meaning to the current task and to the depth of semantic evaluation (Fischler & Bradley, 2006; Schacht & Sommer, 2009). The current interaction consequently indicates that color changed the late evaluative processing associated with positive and negative word meaning. The structure of the present task provides a processing context for this late effect. Participants initially encoded a compound stimulus containing color, direction, and an emotional word, retained these features until the judgment prompt appeared, and then selected the dimension required for the response. These operations engaged sustained evaluation, maintenance of task-relevant information, and response-related updating within the LPC interval. Affective LPP activity and task-related P3b activity can make distinguishable contributions to late positive amplitudes during emotional word processing (Nowparast Rostami et al., 2016). The Color × Valence interaction therefore likely reflects the contribution of both emotional evaluation and task-guided processing to the integration of color and lexical valence. Previous ERP findings support this interpretation. Gan et al. (2016) found that color influenced the processing of moral and immoral words across P200, N300, and LPC stages, showing that learned color-concept associations can shape later evaluation. Xia et al. (2018) similarly found context-specific LPC modulation involving red and green cues, linking late positive activity to sustained attention toward particular color-emotion combinations.

The present interaction is compatible with several related accounts. Color-valence congruency attributes the pattern to the correspondence between learned evaluative meanings of colors and the valence of the words. Affective priming emphasizes the influence of affective information activated by the color cue on the evaluation of lexical meaning. Color-guided attention and associative learning explain how perceptual salience and accumulated experience can alter the allocation of processing resources. A metaphor-related interpretation places these learned associations within a broader conceptual system linking perceptual experience, cultural knowledge, and emotional meaning. ECMT provides one framework for understanding how these sources of information can be weighted according to the immediate linguistic, cultural, and task context. The present Color × Valence interaction is consistent with this context-sensitive interpretation, and the shared predictions of these accounts define the evidential scope of the current result. Designs that independently manipulate metaphorical framing, affective congruency, and cue salience can further distinguish their respective contributions.

The direction of the descriptive color pattern can be understood in relation to learned and culturally situated color meanings. Green is frequently associated with nature, vitality, safety, and positive states, and these associations can support the evaluation of positive emotional material (Clarke & Costall, 2008; Elliot & Maier, 2012). Red carries a broader set of learned meanings. Warning and danger associations coexist with celebration, prosperity, and good fortune in Chinese cultural contexts (Jonauskaite et al., 2020; Bogushevskaya, 2024). This semantic diversity can shape the strength and direction of red-valence relations across participants and situations. ECMT describes metaphorical meaning as an online construction supported by bodily experience, conceptual knowledge, the immediate discourse situation, and cultural context (Kövecses, 2015, 2020). The late color-dependent valence effect is consistent with this contextual construction process. Participant-specific ratings of color-emotion associations in future studies can clarify how culturally shared meanings and individual experience contribute to the LPC pattern.

Spatial direction produced no reliable interaction with word valence. Earlier research has reported vertical-valence congruency when positive and negative concepts are linked to upper and lower spatial locations (Meier & Robinson, 2004; Xie et al., 2014; Y. Zhang et al., 2015). The present study conveyed direction through centrally presented arrows. Such symbols orient attention toward a location and provide an indirect spatial relation (Hommel et al., 2001), which may produce a weaker vertical association than the physical presentation of words in upper and lower visual fields. Color was displayed concurrently and supplied a visually salient affective cue throughout the compound stimulus. Experimental and filler trials were randomly intermixed, and the judgment prompt appeared after the compound stimulus, limiting advance preparation for a particular cue-response relation while keeping color and direction relevant across the task. The null spatial interaction may reflect context-sensitive weighting of the available cues. A direct comparison of single-cue and combined-cue conditions can test the relative weighting and possible competition of color and spatial information.

Bodily and perceptual experience supplies recurring color and spatial associations, conceptual mappings organize these associations in relation to emotional meaning, and contextual information shapes their online activation. The progression from early valence sensitivity through N400 semantic processing to color-dependent LPC evaluation illustrates how perceptual and conceptual information can enter emotional word processing at different times. This sequence also contributes to the study of emotional and cognitive intelligence by identifying neural stages through which contextual cues participate in the evaluation of emotional language. Two features define the current evidential scope. The final ERP sample of 27 participants, comparable with related ERP studies, limits the precision of higher-order interactions and spatial null effects. In addition, symbolic arrows combine directional meaning with attentional orienting, and participant-specific color associations were not measured. The factorial design established whether valence-related effects varied across the concurrently presented color and direction cues. Direct classification of additive integration, competitive interaction, and cue dominance requires single-cue baseline conditions and explicit comparisons between single-cue and combined-cue trials. Future research can use larger samples, direct upper and lower word locations, separate and combined cue conditions, individual association ratings, and cross-cultural comparisons. These extensions can refine the account of how perceptual cues, learned associations, and cultural knowledge jointly shape the time course of emotional word processing.

## 7. Conclusions

The present study investigated how color and symbolic vertical-direction cues modulated the processing of positive and negative Chinese emotional words. Word valence influenced the P1, N1, and N400 components, tracing a temporal progression from early visual-attentional differentiation to lexical-semantic processing. A significant Color × Valence interaction emerged in the LPC window, showing that color information participated in later evaluation of emotional word meaning. Interactions involving spatial direction were nonsignificant across the four components. This temporal pattern identifies early and sustained sensitivity to lexical valence and a later stage of color-dependent contextual modulation.

The findings are compatible with a context-sensitive account of emotional word processing informed by embodied cognition and Extended Conceptual Metaphor Theory. Perceptual experience supplies recurring associations among color, space, and emotion, conceptual mappings organize these associations, and immediate task conditions and cultural knowledge shape their online contribution to meaning construction. The LPC interaction was characterized by a larger positive-minus-negative difference under green cues and a smaller difference in the opposite direction under red cues. This interaction provides evidence that the evaluative contribution of color varied with word valence during late processing. Symbolic vertical-direction cues produced no reliable modulation in the present paradigm, suggesting that the contribution of spatial information may depend on cue format, associative strength, and the configuration of concurrent perceptual information.

The final ERP sample of 27 participants, comparable with related ERP studies, defines the precision of the present higher-order interaction estimates and spatial null effects. The use of centrally presented arrows also combined symbolic directional meaning with attentional orienting. Future research can increase sample size, present words directly in upper and lower visual fields, compare single-cue and combined-cue conditions, collect participant-specific color-emotion association ratings, and examine culturally diverse groups. These extensions can clarify the relative contributions of perceptual salience, learned association, cultural meaning, and metaphor-related mapping. The current findings provide a temporally resolved account of how lexical valence and perceptual context jointly contribute to Chinese emotional word processing.

## Figures and Tables

**Table 1:** Descriptive statistics and matching results for the emotional word stimuli.

Measure	Group	M (SD)	t (df)	*p*	d
Valence	P	6.83 (0.31)	68.93 (158)	<.001	10.64
	N	3.07 (0.39)			
Arousal	P	5.54 (0.60)	−1.77 (158)	.079	0.27
	N	5.70 (0.61)			
Stroke count	P	17.80 (3.02)	1.34 (158)	.182	0.21
	N	17.21 (2.61)			
Word frequency	P	4.53 (0.40)	0.51 (158)	.611	0.08
	N	4.50 (0.38)			
Familiarity	P	6.18 (0.72)	0.69 (158)	.493	0.11
	N	6.10 (0.75)			
Concreteness	P	5.43 (0.81)	0.61 (158)	.541	0.10
	N	5.35 (0.84)			

Note. Word frequency values were log10-transformed before analysis. M = mean; SD = standard deviation; P = positive words; N = negative words.

## Data Availability

The data presented in this study are available on reasonable request from the corresponding author. The data are not publicly available due to privacy and ethical restrictions related to participant EEG recordings.
